# Bluegrass Biodesign: Why an Integrated Biomedical Engineering Curriculum is Crucial for Medical Education

**DOI:** 10.7759/cureus.47261

**Published:** 2023-10-18

**Authors:** Ankur Gupta, Danh Tran, Daniel Nguyen, Elizabeth Bridwell, Hanna Thompson, Faizan Ahmed, Jennifer K Brueckner-Collins, Hermann Frieboes, In Kim, Beth Spurlin

**Affiliations:** 1 Department of Medical Education, University of Louisville School of Medicine, Louisville, USA; 2 Department of Emergency Medicine, Duke University, Durham, USA; 3 Department of Anatomical Sciences and Neurobiology, University of Louisville School of Medicine, Louisville, USA; 4 Department of Bioengineering, University of Louisville, Louisville, USA; 5 Department of Pediatric Emergency Medicine, University of Louisville School of Medicine, Louisville, USA

**Keywords:** biodesign curriculum, healthcare technology, transdisciplinary engineering education, transdisciplinary medical education, biodesign innovation, biomedical engineering, biodesign, innovation, medical education

## Abstract

Background

Medical education often overlooks the significance of design and innovation literacy, resulting in a knowledge gap in undergraduate medical education (UME) regarding formal training in these areas. Incorporating innovation into UME’s core curriculum is crucial as future physicians will encounter evolving technologies, and fostering a transdisciplinary approach can enable collaborative problem-solving and improve patient health outcomes.

Methodology

We developed a comprehensive medical biodesign curriculum focused on innovation, including problem identification, prototype testing, and product commercialization. Participants were selected based on applications, interviews, and diverse criteria. A survey was conducted before and after the program to assess students’ biodesign experiences and knowledge, with data analyzed using descriptive statistics and paired t-tests.

Results

Of the 41 participants, 24 (58.5%) completed both pre- and post-program surveys. These five-point Likert surveys showed a significant shift from pre-program to responses demonstrating increased “comfort levels in explaining and applying biodesign principles” (p < 0.0001). Specifically, the “comfort level in taking a product to market” increased from 33% to 67% (p = 0.01), while the “comfort level in applying the biodesign process” increased from 29% to 92% (p < 0.0001). Moreover, 58.3% of participants expressed interest in continuing their current projects, and 70.8% of students stated feeling confident in generating ideas and solutions with their team members.

Conclusions

The medical biodesign curriculum demonstrated success in exposing undergraduate medical and engineering students to the concepts of medical innovation and biodesign. The program has led to a significant improvement in students’ knowledge and comfort levels in applying the biodesign process and taking a product to market. The high level of interest and participation in the program highlight the need for incorporating innovative training in UME to foster creativity and prepare future physicians to contribute to the advancements in healthcare.

## Introduction

Medical devices are an integral part of daily clinical practices across all medical specialties. The impact of device innovation on the medical field may be far-reaching, including improvement in patient safety, treatments, and reduction in time and costs. However, formal training in innovation and design is rare in medical education. Notably, undergraduate medical education (UME) has a history of encouraging adaptability within healthcare, promptly restructuring the objectives to ensure that students are well-equipped with the knowledge needed to provide high-quality care [1-3]. The implementation of such fundamental changes requires standardization that may often be stagnated by a lack of enthusiasm. This is particularly evident in the field of biomedical innovation and design where formal technological education for students is minimal, resulting in a knowledge gap within medical education that overlooks the significance of design and innovation literacy.

Future physicians will likely encounter the constant evolution of new technologies in patient care. To grow alongside this shift, it is crucial that UME begin to incorporate these aspects of innovation into the core curriculum. Recently, there has been a rise in interest for medical students to obtain experience in innovation, the process of biodesign, and entrepreneurship [4]. As a result, several programs have been developed to meet those needs [1]. One of the earliest biodesign innovation programs is the Stanford Biodesign Innovation Program™ [5]. First introduced in 2001, the program was designed to provide fellows with an opportunity to work in teams to identify unmet clinical needs, develop and prototype medical devices, and launch products into the market.

Although the number of programs has increased, few emphasize the significance of a transdisciplinary approach [1]. In the traditional model of medical education, there are limited opportunities for students to collaborate in transdisciplinary teams working collectively toward the common end goal of enhanced patient health [6,7]. However, breakthroughs in innovation are a result of strong collaborative efforts among those from different areas of expertise [8,9]. Through a transdisciplinary team, utilizing medical and engineering groups, this approach can cut across disciplinary lines and investigate questions and solutions that exist at the intersection of their respective fields. A training program that incorporates transdisciplinary collaboration as a core principle has the potential to help train future physicians to work and lead effectively in diverse teams [4].

Often, physicians are able to identify unique problems in healthcare but may lack the expertise needed to develop their ideas into concrete solutions [10,11]. Alternatively, engineering students have the expertise to solve such problems but lack the medical experience and exposure to the hospital setting needed to fully understand these issues. To address this gap, it is crucial that students are exposed to collaborative environments early in their training to nurture and refine new skillsets that are necessary for such an endeavor [12].

Bluegrass Biodesign is a student-led medical biodesign initiative housed in the University of Louisville School of Medicine and works closely with the University of Louisville Speed School of Engineering. It is a biomedical incubator that facilitates collaboration across both schools in an effort to introduce innovation-specific training. The main goals of Bluegrass Biodesign were to (i) provide students with knowledge and experience in innovation and (ii) cultivate a culture of collaboration within Louisville by inspiring students from different fields to collaborate on the shared goal of improved patient health.

## Materials and methods

This study was approved by the Institutional Review Board (IRB) at the University of Louisville (IRB # 22.0530).

Curriculum

We designed a curriculum aimed at providing a comprehensive foundation to enhance participants’ knowledge of several aspects of innovation, including problem identification, prototype testing, and product commercialization. The structure of the curriculum was centered on lectures and workshops that aimed to standardize the educational aspect of biodesign (Table 1).

**Table 1:** Bluegrass Biodesign curriculum timeline. Curriculum live lectures and workshop titles. *: Indicates sessions that include lectures followed by an in-person workshop experience.

Phase 1: Identify	
Stage 1: Needs finding	
April	How to identify clinical problems*
	Keeping a good notebook
	Observation and problem identification
Stage 2: Screening and refining	
May	Customer discovery
June	Process of commercialization*
July	Market/Stakeholder analysis
August	Needs statement development*
	Presentation and pitch basics
Phase 2/3: Invent and Implement	
Stage 3: Developing concept	
September	Designing a product*
October	Ethics in innovation*
November	Prototype testing basics*
December	Bluegrass and beyond

Notably, the program covered three distinct phases split into two periods: “Identify” and “Invent/Implement.” The “identify” phase focused on the clinical aspect of problem identification. Specifically, there was an emphasis on developing the skills needed to formulate initial clinical questions that were deemed feasible and have an identifiable need within the hospital setting. During the second component of the “identify” phase, participants refined their initial questions into a comprehensive needs statement. This process finalized their identified problem as they progressed into the next period of the program: invention and implementation. During these phases, emphasis was placed on understanding the mechanistic aspect of innovation as participants began the initial stages of product design. Students were expected to attend in-person/virtual lectures once a month for nine months and ultimately utilize their learning to develop a proof of concept.

Generation of proof of concept

The program encouraged students to actively apply the principles taught in the curriculum by developing a proof of concept that addressed a clinical problem that they had identified. To ensure the successful completion of their projects, students were required to meet specific deadlines at various stages. These milestones included submitting a design report, creating computer-aided design (CAD) drawings of their ideas, and constructing a proof of concept, often as a first-generation prototype. Toward the end of the program, students had the chance to showcase their final projects at the program’s “Pitch Day,” a platform for them to present their accomplishments.

Student selection

**Figure 1:**
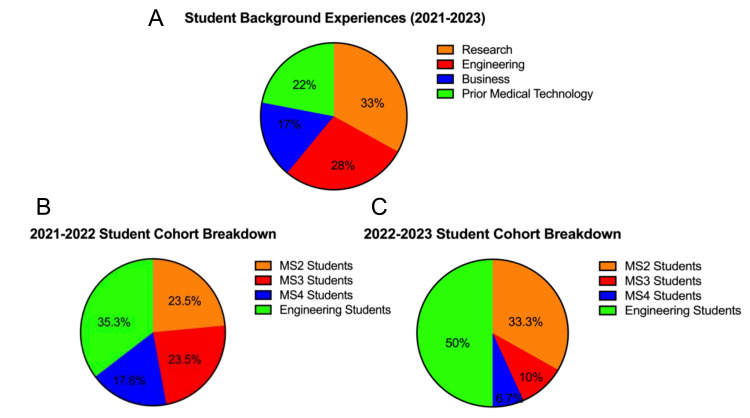
Demographic data among participating students. (A) Student background experiences among all years. (B) Student breakdown in 2021-2022 among MS2 (second-year medical students), MS3 (third-year medical students), MS4 (fourth-year medical students), and engineering students. (C) Student breakdown in 2022-2023.

Participants for Bluegrass Biodesign were selected from rising second-, third-, and fourth-year medical students and undergraduate senior engineering students from the University of Louisville. To be selected, students submitted an application consisting of short-response answers and their curriculum vitae (CV) and underwent interviews with the student and faculty executive board. These interviews focused on communication skills and emotional intelligence. The main criterion for selection is included in our recruitment rubric (Table 2), in which we focused on five different characteristics, namely, time management, past experiences, commitment, creativity, and genuine interest. The result was a culmination of students with a diverse set of experiences and backgrounds (Figure 1A). In the pilot year of the program (n = 17), we had three teams, representing three different medical departments, i.e., otolaryngology, neurosurgery, and cardiology. In the second year of the program (n = 30), we expanded to five teams, adding urology and pulmonology/critical care. Each team comprised three medical students and two or three engineering students (Figures 1B, 1C).

**Table 2:** Student recruitment rubric. Student recruitment rubric based on five traits (time management, past experiences, commitment, creativity, and genuine interest) on a scale of 1-5, with 5 being the highest and 1 being the lowest rank.

	1	2	3	4	5
Time management	The applicant shows limited involvement with activities outside of class, with only variable success in these areas or has a history of unfulfilled commitments	The applicant shows attempts of involvement with some success or little involvement with high success	The applicant shows moderate forms of involvement with moderate to high success	The applicant demonstrates the ability to multi-task between multiple extra activities as well as classes with success in all areas	The applicant demonstrates ability well above expectations with evidence of handling activities with high difficulty as well as time consumption while accomplishing all said tasks (as well as classes) with a high degree of success
Past experiences	The applicant indicates no notable past experiences with no expertise	The applicant indicates a history of no previous involvement in a project but presents with a moderate level of expertise	The applicant indicates a history of previous involvement in assisting with at least one project in which moderate expertise was demonstrated	The applicant indicates a history of previous involvement with starting at least one project in which strong expertise was demonstrated	The applicant indicates a history of previous involvement with starting multiple projections in which exceptional expertise was demonstrated
Commitment	The applicant has no examples of completed projects or commitments	The applicant has one completed project or involvement in projects without completion or an intermittent long-term commitment with minimal effort	The applicant has a small number of completed projects with moderate time commitment or a long-term commitment with moderate to low time consumption	The applicant has examples of multiple time-intensive completed projects or a long-term commitment requiring high time consumption	The applicant has examples of a long-term lifetime/trans-institutional commitment
Creativity	The applicant tends to stick to standardized methodologies	The applicant demonstrates creative tendencies but has not yet develop a unique approach	Applicant shows creative ideas/approaches that are plausible for future commitment.	The applicant shows unique approaches to a field of interest and they deem these approaches as comparable to traditional	The applicant shows unique approaches to multiple fields and they deem these approaches as more successful than traditional
Genuine interest	The applicant shows very limited true interest in the field of biodesign	The applicant shows interest but is unable to articulate reasons for wanting to be a part of the program	The applicant shows moderate levels of interest	The applicant shows high levels of interest with motivations stemming from past experiences	The applicant demonstrates interest with multiple project ideas or a vast amount of experience or knowledge in the field of biodesign

Study design

To assess the effectiveness of the medical biodesign program in achieving its goal of exposing students to the concepts of medical innovation and evaluating its impact on their education, we designed a survey for 41 student participants. These surveys were specifically designed to elicit valuable feedback regarding their experiences within the program, while also enabling us to assess any notable shifts in their knowledge and comfort levels concerning the fields of biodesign and the process of bringing a product to market.

Survey

The study included responses from medical and engineering students between the years of 2021 and 2023. Its objective was to assess the effectiveness of the program in introducing students to the concepts of medical innovation. To gather feedback about their experiences, surveys were created using Google Forms and distributed via email to second-, third-, and fourth-year medical students, as well as senior undergraduate engineering students. Before commencing the program, all participants voluntarily completed a pre-program survey, and upon program completion, they filled out a post-program survey. To assess participants’ understanding of the biodesign process, the surveys included a question asking, “On a scale of 1-5 what is your comfort level with applying the biodesign process in your future practice?” Similarly, knowledge of taking a product to market was evaluated by asking, “On a scale of 1-5 how comfortable are you explaining the process of taking a product idea to the market?” All questions were scored based on a Likert scale of 1 = strongly uncomfortable, 2 = slightly uncomfortable, 3 = neutral, 4 = slightly comfortable, and 5 = strongly comfortable.

Data analysis

Survey data from Google Forms was downloaded to Microsoft Excel and GraphPad Prism for analysis via descriptive statistics. Paired t-tests were done to determine differences in student responses before and following the commencement of the Bluegrass Biodesign program. P-values <0.05 were denoted as being statistically significant data.

## Results

Of 41 medical students, 24 (58.5%) completed both the pre- and post-program surveys, and only those participants who completed both survey components were included in the study. The gender distribution among the participants was 53.2% male and 46.8% female. Additionally, 61% of participants had a background in research and engineering.

**Figure 2:**
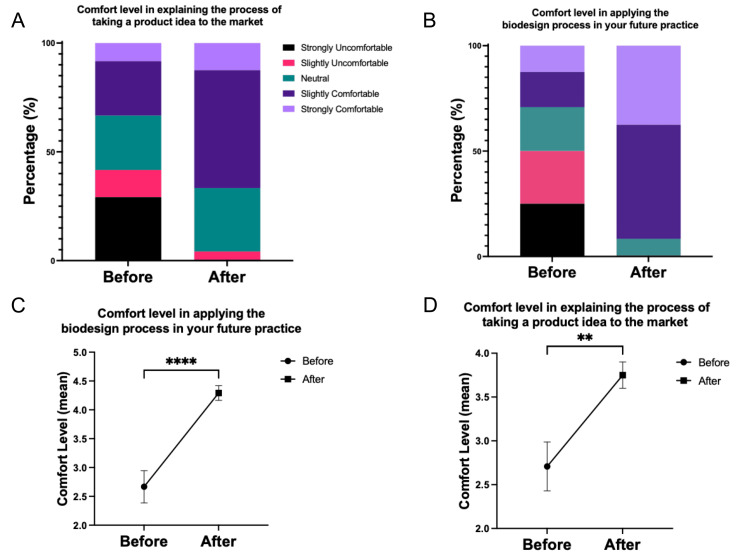
Student comfort levels. (A) Comfort level in explaining the process of taking a product idea to the market using a 1-5 Likert scale. (B) Comfort level in applying the biodesign process in your future practice using a 1-5 Likert scale (1 indicates strongly uncomfortable while 5 indicates strongly comfortable). (C) Graphical visualization of comfort level in explaining the process of taking a product idea to the market before and after completion of the program. (D) Graphical visualization of comfort level in applying the biodesign process in your future practice before and after completion of the program expressed as mean + SEM. n = 24, **p = 0.01, ****p < 0.0001.

The responses from the surveys indicated an improvement in students’ comfort level after their participation in the Bluegrass Biodesign curriculum (Figure 2, Panels A and B). The Likert survey responses shifted from a normal distribution before the program to responses that demonstrated increased comfort levels in explaining and applying biodesign principles to product development, as well as in their future practice. Specifically, the comfort level in taking a product to market increased from 33% of responses above neutral to 67%, and the comfort level in applying the biodesign process increased from 29% to 92%.

Based on survey responses, the self-described improved comfort level was statistically significant for both the knowledge of the biodesign process (p < 0.0001) and taking a product to market (p < 0.01), as shown in Figure 2 (Panels C and D). Notably, the average answer for knowledge regarding taking a product to market increased significantly from 2.70 to 3.75, similar to the improvement observed in the knowledge of the biodesign process, where the average answer increased from 2.67 to 4.29.

**Figure 3:**
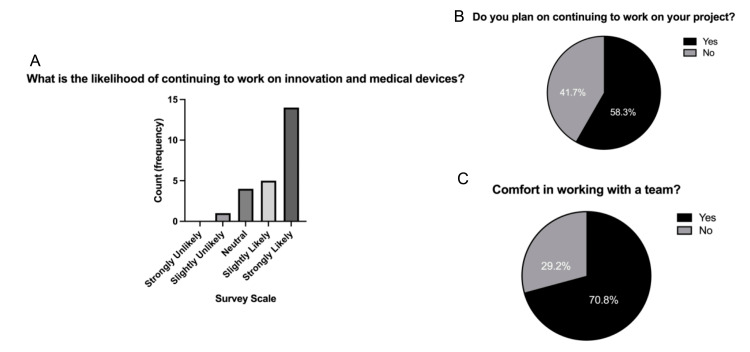
Student survey responses. (A) Survey response for the likelihood of continuing to work on innovation and medical devices. (B) Survey response for planning to continue working on respective projects. (C) Comfort in working with a team survey response.

A majority of the participants expressed a likelihood of continuing to pursue innovation in medical devices after completing the program, as illustrated in Figure 3, Panel A.

Specifically, 58.3% of participants showed interest in continuing their current project following the gained experience in navigating interdisciplinary teams throughout the program (Figure 3, Panel B), and 70.8% of students stated that they felt comfortable generating ideas and solutions with other team members (Figure 3, Panel C). Another noteworthy finding was the increase in the number of applicants from the first to the second cohort. Despite only four additional spots being offered (11 open spots in 2021 vs. 15 open spots in 2023), the number of applicants nearly doubled from year 1 (18) to year 2 (34).

Finally, the success of the program can be measured through the successful completion of the biodesign process, which includes problem identification and innovation of a proof of concept that effectively addresses the identified problem. Over the past two years, each team has successfully identified a solvable and marketable problem, as presented in Table 3.

**Table 3:** Identified problems in Bluegrass Biodesign.

Year 1	
Team	Problem identified
Neurosurgery	Decreasing the time from stroke onset to in-hospital treatment
Otolaryngology	Intraoperative visualization of the parathyroid during thyroidectomy
Cardiothoracic surgery	Decreasing the rate of ventilator acquired pneumonia
Year 2	
Team	Problem identified
Neurosurgery	Improving intraoperative utilization of imaging
Otolaryngology	Preventing iatrogenic laryngeal stenosis
Cardiothoracic surgery	Improving incentive spirometry
Critical care/Pulmonology	Decreasing bronchoscopy complications
Urology	Improving intermittent self-catheterization

## Discussion

Based on the results, there are several strong indicators of the success of Bluegrass Biodesign. The responses to the survey yielded statistically significant improvement in the students’ learning of the biodesign process and taking an idea to market. Additionally, each team successfully identified a clinical problem and built a proof of concept for their proposed solution, and the program has seen steady growth in interest among the student body and, thus, increased program impact.

Interest in incorporating biodesign into medical education

In comparison to our first year, we observed a notable increase in the number of applicants who applied for the program (18 vs. 34). Additionally, due to the expressed interest of several other clinical departments, we were able to increase the number of teams from three to five in year two. The increased involvement of faculty and students alike suggests an interest in learning more about the processes involved in clinical innovation through biodesign. Several previous studies have documented comparable results where students have demonstrated a desire to learn about and participate in collaborative biodesign [4,13]. Part of the allure of a program like this stems from the notion that the conventional UME often lacks sufficient training in creative thinking and entrepreneurship [4,14], including problem identification, multidisciplinary leadership, and resource sharing in its curriculum [4]. As a result, there has been a significant increase in the number of student-led biodesign initiatives in recent years aimed at targeting these unmet needs [1]. Given the growing enthusiasm at both the local and national levels, it suggests that a systematic educational change may align with the interests of future and present medical students and meet the future needs of the rapidly evolving landscape of healthcare.

While the need for medical innovation is often met with general agreement, the establishment of a concrete curriculum dedicated to needs finding, market analysis, stakeholder analysis, and prototyping is also often dedicated to graduate medical education with little implementation in medical schools. Similarly, many medical biodesign programs follow the framework created by Stanford BioDesign, which consists of physicians, engineers, and business members from the graduate levels of education. As technology in medicine continues to progress, it becomes increasingly clear that earlier engagement in the medical education pipeline, specifically undergraduate medical students, may benefit the process of medical innovation.

The desire to innovate is innate in many medical students, with many communicating their desire to be involved in the progression of science as a reason for pursuing medicine as a career. To meet this need, medical schools are starting to adapt their curricula to create space for creativity and innovation. The purpose of Bluegrass Biodesign was to help fill the gap that exists in knowledge surrounding innovation in medicine at the UME level. Through the creation of multidisciplinary teams that approach clinical problems in a stepwise model, medical students had the opportunity to gain first-hand experience in the steps.

Our survey results demonstrated that providing biomedical innovation training to medical students can prove to be valuable in encouraging students to understand and pursue knowledge in this domain. One of the intriguing findings was that most students did not have prior knowledge in the field of biomedical innovation and had an interest in learning more. It was encouraging to observe that medical students expressed continued interest in the field and a desire to implement their knowledge of biodesign and innovation in their future careers.

Major program takeaways

The demands of clinical rotations and board preparation added challenges, affecting communication between faculty, medical students, and engineering students. As the program progressed, engagement in the form of lecture attendance and communication between student team members and faculty diminished. Part of the rationale for the observed trend may be a result of the academic schedule in which there were more exams later in the academic semester. Given that Bluegrass Biodesign started at the beginning of the summer and concludes after the end of the winter semester, it was reasonable to expect less focused attention as educational obligations increased.

The program’s curriculum also acted as a point for improvement. The program’s foundation centered on three main concepts, namely, identify, invent, and implement. Currently, the curriculum focuses on identification and invention, leaving room for growth in the “implement” portion of the program, specifically the marketing and business aspects of innovation. Integration with the University of Louisville business school continued to be a point of development within the program’s curriculum which is a goal with similar national biodesign programs [15].

Limitations

Our study has several limitations. First, the relatively small sample size limits the generalizability of the findings to a broader population of medical and engineering students. Although our study is still in the proof-of-concept stage, we hope to collect data from future cohorts to reinforce the evidence for the effectiveness of biodesign education in medical school curricula. Second, our short-term evaluation immediately after program completion does not capture the long-term effects of the intervention on participants’ careers and contributions to healthcare innovation. We will gain a better understanding of the impact of our curriculum and program on these cohorts as they go into the commercialization phase for their respective projects. Finally, the reliance on self-reported data in our surveys may introduce social desirability bias. While we took steps to mitigate this by having students complete the surveys anonymously and encouraging them to provide honest feedback, it is important to be aware of this potential limitation.

Future directions

Our future goals revolved around a comprehensive expansion of the program, which included diversifying the range of departments involved. While our program currently has a strong emphasis on surgical departments, we aimed to broaden our scope to incorporate non-surgical specialties as well to allow for a more diverse and inclusive approach to innovation and problem-solving. We anticipated significant growth in participating specialties and subsequently formation of additional teams, allowing increased student involvement.

Regarding our curriculum, we were committed to incorporating business-oriented lectures into our program. These lectures focused on areas such as commercialization, total addressable market, and creating a business plan. While our program was not directly integrated with the business school, we have established collaborations with the School of Business.

In our third cohort, Bluegrass Biodesign prioritized addressing diversity and health inequities through the conscientious application of biodesign principles. Aligning with the University of Louisville School of Medicine’s emphasis on cultural competence and healthcare disparities, we planned to host expert-led lectures on these important subjects. Additionally, we aimed to employ faculty experienced in addressing health equity via innovative solutions and encourage student projects focused on these issues. Such initiatives underlined the importance of benefiting underserved populations and aligned with the University’s equity-driven mandate.

## Conclusions

The integration of a transdisciplinary biomedical program such as Bluegrass Biodesign may be crucial for medical education today. The traditional model of UME often does not adequately address the need for formal training in innovation, design, and entrepreneurship. By incorporating innovation and design literacy into the core curriculum, medical students may develop the competency needed to navigate and contribute to the constant emergence of new technologies in healthcare. The growing interest in programs such as Bluegrass Biodesign, as indicated by the increase in recruitment numbers and programs across the country and the desire of students to continue working on innovation and medical devices, highlights the importance of incorporating innovation and design education in medical curricula.
